# BDNF Val66Met Variant and Smoking in a Chinese Population

**DOI:** 10.1371/journal.pone.0053295

**Published:** 2012-12-28

**Authors:** Xiang Yang Zhang, Da Chun Chen, Mei Hong Xiu, Xingguang Luo, Lingjun Zuo, Colin N. Haile, Therese A. Kosten, Thomas R. Kosten

**Affiliations:** 1 Menninger Department of Psychiatry and Behavioral Sciences, Baylor College of Medicine, Houston, Texas, United States of America; 2 Psychiatric Research Center, Beijing Hui-Long-Guan hospital, Peking University, Beijing, China; 3 Department of Psychiatry, Yale University, New Haven, Connecticut, United States of America; Instituto de Higiene e Medicina Tropical, Portugal

## Abstract

Several recent studies have supported the hypothesis that brain-derived neurotrophic factor (BDNF), a member of the neurotrophic factor family, might be associated with nicotine addiction. Association studies have also suggested that the BDNF gene might play a role in the susceptibility to nicotine dependence but results appear contradictory. The present work was therefore undertaken to examine the association of smoking with the BDNF *Val66Met* gene polymorphism in Chinese population. The BDNF *Val66Met* gene polymorphism was examined in 628 healthy male volunteers including 322 smokers and 306 non-smokers. Also, the BDNF serum levels were measured in 136 smokers and 97 nonsmokers. Our results showed no significant association between the BDNF *Val66Met* polymorphism or serum levels among smokers and non-smokers. Smokers with the *Met* allele however started smoking significantly earlier than those with the *Val/Val* genotype (mean age at smoking initiation of 17.4, 17.9 and 21.2 years for *Met/Met*, *Met/Val*, and *Val/Val*, respectively; both p<0.05). No other significant differences between other variables such as number of cigarettes per day, smoking severity as measured by the Fagerstrom Test for Nicotine Dependence (FTND) score and carbon monoxide (CO) levels (all p>0.05). In addition, there was no main effect of genotype on serum BDNF levels. Our findings suggest that the BDNF *Val66Met* polymorphism may not be involved in susceptibility to smoking among the Chinese male population, but may influence the age at which smoking is initiated. However, the findings must be interpreted with caution because of the relatively small sample size for an association study. Results should be confirmed in a larger cohort.

## Introduction

Smoking is influenced by genetic and environmental factors. Genome-wide association studies (GWAS) have identified genetic variation associated with smoking behaviors, including smoking initiation (SI), smoking quantity and smoking cessation (SC) [1] (David et al 2012). Several large GWAS of smoking quantity identified associations with genetic variants in the nicotinic acetylcholine receptor α5, α3 and β4 subunit cluster on chromosome 15q25.1 in populations of European ancestry [2]–[5]. Also, the most recent genome-wide association study meta-analysis of smoking behavior in African Americans confirmed this region as an important susceptibility locus for smoking quantity (cigarettes per day) in men and women of African ancestry [1].

Molecular epidemiological studies implicate genetic factors in the etiology of smoking and nicotine dependence including genetic influence in the dopamine (DA) reward pathway, which is activated by nicotine [6]. Preclinical studies have demonstrated that nicotine activates dopaminergic neurons in the mesolimbic reward pathway and enhances DA release [7]. Since DA plays an important role in nicotine dependence, various dopamine-related genes that influence DA modulating peptides are plausible candidates that may contribute to increased risk for developing nicotine dependence [8]. Recent studies suggest brain-derived neurotrophic factor (BDNF) to be a potent DA modulating peptide, that may regulate nicotine reward [9], [10].

BDNF, a member of the neurotrophic factor family, is widely expressed in the adult mammalian brain and plays a critical role in the development, regeneration, survival and maintenance of neuronal function [11]. BDNF is essential for normal DA neuron growth and function [12]–[14]. Specifically, BDNF is involved in the proper neurodevelopment and regulation of dopaminergic-related systems [12], [13], [15]. BDNF is also neuroprotective [16]. These findings suggest a potential role of BDNF in functional activity of DA neurons during neurological insult. BDNF appears to be essential for normal expression of the DA D3 receptor in nucleus accumbens both during development and in adulthood [17]. That BDNF may influence DA responsiveness in such an important manner suggest that it may relate in some way to the etiopathology and/or treatment of several conditions implicating DA [17]. One recent study showed a modulatory role for BDNF genetic variation on genetically mediated differences in the mesolimbic dopaminergic system in the context of human personality [18].

Previous studies show that acute nicotine administration significantly decreases BDNF mRNA whereas chronic nicotine, but not other drugs of abuse (i.e., cocaine or morphine) increase BDNF in the rat hippocampus [19]. Genome-wide linkage scans indicate that the region of chromosome 11p13 where the BDNF gene is located likely harbors susceptibility genes for nicotine dependence [20]. A similar study implicated BDNF variants in vulnerability to abuse multiple drugs [21]. Moreover, the lower plasma concentration of BDNF was reported in the smoking group [22].

A single nucleotide polymorphism (SNP) that determines a valine-to-methionine variation at codon 66 of the BDNF coding sequence (rs6265) has been implicated in human memory and hippocampal function [23]. Although this polymorphism does not affect mature BDNF protein function, it alters intracellular trafficking and packaging of pro-BDNF and consequently, secretion of the mature peptide [23], [24]. The evidence suggests an association between allelic variants of BDNF and nicotine dependence in male European-American smokers [24]. A recent study demonstrated that the frequency of both *Met/Met* genotype and Met allele was significantly increased in current and in former smokers when compared to never smokers among Germany population, suggesting that carries of the BDNF *Met* have an increased risk for smoking [9]. However, a subsequent association study did not corroborate these findings [10]. Recent studies however, have linked this BDNF polymorphism with smoking initiation [25] and smoking cessation [26]. Thus, studies are necessary to better define the role of BDNF *Val66Met* in nicotine dependence and whether harboring this polymorphism may have therapeutic implications.

The present work was undertaken to examine the association between smoking and the BDNF *Val66Met* gene polymorphism or serum BDNF levels in an independent sample from China. The *a priori* study goals and hypotheses were that the *Val66Met* polymorphism in the BDNF gene might influence risk for smoking or be associated with smoking phenotypes in healthy human controls of Chinese descent. In China, female smoking is extraordinarily rare in general population (male/female: 67.1% vs. 7.1%) [27], thus we focused on male subjects in this study.

## Materials and Methods

### Ethics statement

After a complete description of the study, all subjects gave their written informed consent to participate in the study. The protocol was approved by the Institutional Review Board (IRB), Beijing HuiLongGuan hospital.

### Subjects

Six hundred and twenty-eight healthy unrelated male volunteers (aged 22–70 years, mean age: 46.3±12.7 years) were recruited from the Haidian district in Beijing. The resident registration files provided a random sample of control subjects, and we sent each subject a letter explaining the purpose of the study. Of the 1015 eligible subjects, 679 completed the baseline interview (participation rate: 66.9%) from June, 2006 to December, 2007. This was an independent study sample, different from our previous work [28]. Local officials and health centers arranged for the interviews and measurements to take place at the center office at times convenient to the participants. The participants, who were of Chinese Han descents, were interviewed by trained investigators, using a detailed questionnaire including general information, sociodemographic characteristics, current and previous smoking behavior, and medical and psychological conditions. A clinical interview was used to exclude potential controls with Axis I disorders by a research psychiatrist.

The subjects were divided into groups based on their smoking history. *Nonsmokers* were defined as individuals who had smoked less than 100 cigarettes during their lifetime. *Former smokers or quitters* were defined as persons who had previously smoked more than one cigarette each day but had quit smoking for more than 1 year. *Current smokers* were defined as persons who smoked more than one cigarette each day and have smoked for more than 1 year. Age at smoking initiation was identified as age of initiating regular or daily smoking.

If the subjects identified themselves as a smoker, then further questions determined the average number of cigarettes per day in 1 week before entry into the study. If the subject was currently a non-smoker, further questions were asked regarding previous smoking behavior including whether or not they had quit smoking. Quitters or ex-smokers were excluded from the present study, since there was comparatively less number of quitters in the present study (n = 51).

In addition, the Chinese translation of the standardized Fagerstrom Test for Nicotine Dependence (FTND) was employed to measure the degree of ND [29]. Additional visits were requested for subjects with missing or ambiguous data. Exclusion criteria included a history of ―Diagnostic and Statistical Manual of Mental Disorders—Fourth Edition ‖ (DSM-IV) psychiatric disorder, alcohol abuse, and other drug abuse. Demographic data are summarized in Table 1.

**Table 1:** Characteristics of smokers and non-smokers.

Category	Smokers	Nonsmokers
	N = 322	N = 306
Age (years)	46.6±11.8	45.8±13.3
Education (years)	9.1±3.5	9.2±3.6
BMI (Kg/m^2^)	25.4±6.1	24.9±5.8
BDNF(ng/ml)^a^	11.9±2.4	11.6±3.3

Note: BMI = body mass index; BDNF = brain-derived neurotrophic factor.

a BDNF serum level data were available from 136 smokers and 97 nonsmokers.

### Genotyping procedure

DNA was extracted using standard protocols. The genotypes of the BDNF Val66Met polymorphisms were identified as reported in our previous study [30]. A research assistant who was blinded to the clinical status genotyped every subject twice for accuracy of genotyping.

### Serum BDNF measurement

Fast serum BDNF levels were measured by sandwich ELISA using a commercially available kit. A full description of the assay has been given in our previous report [31]. All samples were assayed by a research assistant blind to the clinical situation. Inter- and intra-assay variation coefficients were 7% and 5%, respectively.

### Measurement of carbon monoxide

Measurement of carbon monoxide (CO) concentrations in exhaled air was performed using BreathICO (Vitalograph, Buckingham, UK). Participants were instructed to hold their breath for 15 s and then exhale into a disposable tube attached to the BreathICO. Breath CO concentrations were expressed in parts per million (ppm).

### Statistical analysis

The Hardy-Weinberg equilibrium in the smokers and non-smokers was tested by using the x^2^ test for goodness of fit. The x^2^ tests and Fisher's exact test, if necessary, were performed to assess the difference in genotype and allele frequencies in smokers and non-smokers. Odds ratio (OR) and their 95% confidence intervals were calculated to evaluate the effects of different genotypes. Between-group differences in continuous variables were evaluated using the Student's t-test or one-way analysis of variance (ANOVA), followed by the Fisher's least significant difference (LSD) multiple range test for between-group comparison. Bonferroni corrections were applied to adjust for multiple testing. When we found a significant difference between them, we formally assessed whether heterozygotes (*Val/Met*) had an intermediate level by using a linear multiple regression test. Demographic and genotype data were analyzed by using the Predictive Analytics Software (PASW; formerly SPSS Statistics) (SPSS Inc, Chicago, IL, USA). The significance criterion was set at p<0.05.

After determination of the ‘relative risk’ and ‘risk factor's frequency’ in our samples, power estimates (power defined as the chance that true differences will actually be detected) was calculated with Quanto Software [32].

## Results

Table 1 lists subject demographics and characteristics in smokers and non-smokers included in the present study. There were no significant differences in age, education and body mass index (BMI) between two groups (all p>0.05). Allele frequencies, genotype distributions and the statistical analysis are shown in Table 2. Genotype distributions had no deviation from Hardy-Weinberg equilibrium in both groups (both p>0.05). Allelic frequency of BDNF did not differ in smokers compared to nonsmokers (x^2^ = 1.11, df = 1, p = 0.29; OR of 1.13, 95% CI 0.91–1.41). Genotype distribution was also not significantly different between smokers and nonsmoker (X^2^ = 2.26, df = 2, p = 0.32).

**Table 2:** Allele and genotype frequencies of BDNF-Val66Met polymorphism for smokers and non-smokers.

	Genotype				Allele			
	Val/Val	Val/Met	Met/Met	P-value	Val	Met	P-valule	OR (95% CI)
Smokers	81	177	64	(2 d.f.)	339	305	(1 d.f.)	1.13(0.91–1.41)
N = 322	(25.1%)	(55.0%)	(19.9%)		(52.6%)	(47.4%)		
Non-smokers	74	156	76	0.32	304	308	0.29	
N = 306	(23.8%)	(50.0%)	(26.2%)		(49.7%)	(50.3%)		

We also examined the characteristics of smokers based on genotype grouping (Table 3). Among smokers, individuals with *Met* allele started smoking significantly earlier than those with the *Val/Val* genotype (17.4±5.0 years for *Met/Met*; 17.9±5.3 years for *Met/Val*; 21.2±6.4 years for *Val/Val*; both p<0.05). Using multiple regression, the number of BDNF *Met* alleles (0,1,2) was parametrically related to age of smoking onset after adding age, education and BMI to the regression analysis (r^2^ = 0.09, p<0.01). BDNF *Val66Met* genotype accounted for 9% of the variance in age of smoking onset. However, there was no a significant difference in other variables, including the number of smoking cigarettes each day, the FTND score and CO levels (all p>0.05). In addition, given that the primary finding was for age at onset of smoking, the former smokers (n = 51) were also included in the analysis. Further analysis showed that individuals with *Met* allele, started smoking significantly earlier than those with the *Val/Val* genotype (17.1±5.2 years for *Met/Met*; 17.8±5.6 years for *Met/Val*; 21.5±6.8 years for *Val/Val*; both p<0.05). However, although including former smokers provided the same information on age at onset of smoking, this finding did not still maintain significance following correcting for multiple testing (both p>0.05). In addition, excluding those smokers who were obese, the smokers with the *Met* allele still started smoking significantly earlier than those with the *Val/Val* genotype (both p<0.05), and no significant difference in other variables, including the number of smoking cigarettes each day, the FTND score and CO levels was observed in the BDNF *Val66Met* genotype (all p>0.05).

**Table 3:** Smokers characteristics subgrouped according to BDNF Met66Val genotype.

Genotype	Val/Val	Val/Met	Met/Met
	(n = 81)	(n = 177)	(n = 64)
Age (years)	47.3±12.6	45.6±12.3	48.3±10.5
Education (years)	8.7±3.3	9.4±4.5	8.6±3.7
BMI (Kg/m^2^)	25.6±5.4	25.1±6.9	26.1±4.8
Cigarettes/day	14.7±12.9	15.4±12.5	15.8±13.8
Age at smoking	21.2±6.4	17.9±5.3*	17.4±5.0*
FTND score	3.3±3.0	3.9±2.9	4.0±3.4
CO (ppm)	8.1±8.3	7.9±6.7	8.6±6.9

Note: BMI = body mass index; FTND = Fagerstrom Test for Nicotine Dependence; CO = carbon monoxide.

Met allele subgroups versus Val/Val subgroup:

* p<0.05.

This total sample had 0.94–0.99 power to detect dominant, recessive and log additive polymorphic inheritance in smokers with an odds ratio (OR) of 2 or greater (alpha = 0.05, two tailed test).

In addition, serum BDNF levels were available from 136 smokers and 97 nonsmokers. BDNF levels were not significantly different between smokers and nonsmokers (F = 0.34, *p* = 0.56; Table 1). Further, there was no main effect of genotype on serum BDNF levels (11.8±2.6 ng/ml for Val/Val, 11.9±2.7 ng/ml for Val/Met, and 12.0±1.9 ng/ml for Met/Met; F = 0.14, *p* = 0.88). Furthermore, no significant correlation was observed between BDNF levels and age, education, age of smoking onset, the number of cigarettes per day, and body mass index (BMI) (all p>0.05). Further analysis showed that there was no effects of the BDNF level×genotype interaction on smoking status, including age of smoking onset, the number of smoking cigarettes each day, the FTND score and CO levels (all p>0.05). In addition, excluding those who were obese, BDNF levels were still not significantly different between smokers and nonsmokers (p>0.05), and BDNF levels were not associated with age, education, age of smoking onset, the number of cigarettes per day, and body mass index (BMI) (all p>0.05).

## Discussion

To our knowledge, this is the first study to investigate any association between BDNF gene polymorphism and smoking status in a Chinese population. Results revealed no evidence of association between BDNF *Val66Met* polymorphism and smoking among male Chinese. This finding is consistent with one recent study [10], but not with two other studies that assessed the BDNF *Val66Met* polymorphism and smoking in European-Caucasians [9], [24]. However, the BDNF *Val66Met* polymorphism was associated with age at smoking initiation among Chinese male smokers. Smoking behavior was found to be earlier in those carrying the *Met* allele of BDNF *Val66Met* polymorphism. This significant difference did not pass Bonferroni correction however due to multiple testing, suggesting that BDNF *Val66Met* polymorphism may have a small effect.

There are several factors that may have influenced our inability to identify any robust associations between the BDNF *Val66Met* polymorphism and smoking in our experimental population. First, it is possible that our failure to demonstrate a significant association may reflect false negative results due to the modest sample size. Therefore, the association between the BDNF *Val66Met* polymorphism and smoking in our sample should be interpreted with caution until replicated in a larger sample. Secondly, it is possible that additional variants of the gene may influence smoking initiation and the subsequent development of nicotine dependence. For example, haplotype analysis including the SNPs rs6484320, rs988748, rs2030324 and rs7934165 revealed an association between the major T-C-T-G haplotype and smoking are associated in males [24]. Whether these variants contributed to the results in the present study is unknown. Further analysis examining this possibility is warranted.

To our knowledge, this is the first report showing an association between the BDNF gene and age at which smoking was initiated. There are several explanations regarding the possible mechanism(s) attributed to the BDNF polymorphism that may have relate to influencing the age at which individuals start smoking. This polymorphism is located in the 5′ pro-BDNF sequence, which encodes the precursor peptide (pro-BDNF) that is proteolytically cleaved to form the mature protein [33]. Recently, Egan and colleagues reported that this BDNF polymorphism dramatically alters intracellular trafficking and packaging of pro-BDNF and, thus, regulates secretion of the mature peptide [23]. This *Met* variant results in inefficient trafficking of BDNF to secretory granules and reduced activity-dependent BDNF release which impairs hippocampal-dependent memory [23], [34]. These findings suggest that regulation of BDNF secretion and protein production is influenced by the BDNF *Val66Met* gene polymorphism. It is well known that dopaminergic neurons in the mesolimbic area may play a critical role in mediating the reinforcing properties of addictive drugs, including nicotine [35], [36]. BDNF significantly influences DA neurotransmission [15], [17], including synaptic plasticity related to drug reinforcement [37] and abuse [38].Thus, it is likely that the *Val66Met* polymorphism of BDNF alters BDNF gene products, which in turn could affect central DA signaling pathways and alter the behavioral effects of nicotine. However, we did not find the effect of BDNF *Val66Met* genotype on serum BDNF levels. Based on the present data, we are unable to explain why the BDNF *Val66Met* polymorphism was significantly associated with age at which smoking was initiated but not with susceptibility to smoking itself or smoking severity (as measured by the FTND). One possibility however is that the association between age at which smoking was initiated and BDNF *Val66Met* polymorphism is due to linkage disequilibrium between this polymorphism and a functional polymorphism in or near the BDNF. Accordingly, further studies on the association between age of smoking initiation and the polymorphisms in or near BDNF gene may help to clarify the roles of the locus in age of smoking initiation, especially the polymorphisms in the promoter region of the BDNF gene, such as C-281A, which was found to be associated with age of onset of schizophrenia [39]. Still, the magnitude of the effects of BDNF *Val66Met* polymorphism on age of smoking onset was small and correlation coefficients suggest that this polymorphism accounts for approximately 9% of the variance in age at smoking onset in this study group.

In summary, the present study demonstrates that the BDNF *Val66Met* polymorphism influence the age of smoking initiation, but not smoking itself and smoking severity in Chinese male smokers. Although the exact role in which the BDNF *Val66Met* genotype may influence smoking onset are unknown, our results do suggest that men with the variant allele initiated smoking earlier, suggesting a that those harboring this allele may be at risk. That is, this BDNF polymorphism could enhance the reinforcing effects of nicotine in some way. This conclusion is somewhat supported by BDNF's role in other drug dependencies [37], [38].However, studies directly assessing whether the subjective reinforcing effects are altered in those with the BDNF *Val66Met* polymorphism are needed to adequately address this question. Nevertheless, replication and extension of the current findings that BDNF provides a candidate locus for smoking initiation could open the way for both improved understanding and enhanced preventative therapies aimed at those that carry a certain genetic variants of a polymorphism.
